# Disruption of a GalR2–mitochondrial axis in the ventral hippocampus contributes to depression-like phenotypes after prenatal stress

**DOI:** 10.3389/fpsyt.2026.1809262

**Published:** 2026-06-08

**Authors:** Jingjing Yue, Jing Zhang, Xiaoyi Yu, Zhiheng Li, Yanhua Wang, Siyi Zhao, Yunfei Bai, Xiaoxiao Li, Hui Li, Yutao Yang, Zhi-Qing David Xu

**Affiliations:** 1 Department of Neurobiology, Beijing Key Laboratory of Neural Regeneration and Repair, Beijing Institute of Brain Disorders, Capital Medical University, Beijing, China; 2 Department of Pathology, Capital Medical University, Beijing, China; 3 Department of Anatomy and Histology, Capital Medical University, Beijing, China

**Keywords:** depression, galanin receptor 2, mitochondrial quality control, prenatal stress (PS), ventral hippocampus

## Abstract

**Background:**

Prenatal stress (PS) is a major risk factor for depression later in life, yet the cellular mechanisms linking early-life adversity to long-term affective vulnerability remain incompletely understood. Neuropeptide receptors have emerged as important modulators of stress-related psychopathology, but their roles in mitochondrial regulation within limbic circuits remain largely unexplored.

**Methods:**

A rat model of PS was established to assess depression-like behaviors in adulthood. Mitochondrial ultrastructure, ATP production, and the expression of Galanin receptor 2 (GalR2) and key components of the PINK1/Parkin mitochondrial quality control machinery were examined in the ventral hippocampus (vHPC). The effects of intranasal administration of the GalR2 agonist AR-M1896 on behavioral and mitochondrial alterations were evaluated *in vivo*. To directly test whether the vHPC mediates these effects, we performed unilateral intra−vHPC infusion of AR−M1896. *In vitro*, glucocorticoid exposure and pharmacological manipulation of GalR2 were used to assess their impact on mitochondrial function and PINK1/Parkin signaling.

**Results:**

PS induced persistent anhedonia-like behavior and behavioral despair phenotypes in adult offspring, accompanied by marked mitochondrial structural abnormalities, reduced ATP production, and downregulation of GalR2 and PINK1/Parkin-associated mitochondrial quality control signaling in the vHPC. Intranasal AR-M1896 partially normalized reward-related behavioral deficits and ameliorated mitochondrial dysfunction. Importantly, direct intra−vHPC infusion of AR−M1896 elevated ATP, PINK1 and Parkin levels in the ipsilateral vHPC, providing causal evidence that the vHPC is a critical site for GalR2−mediated PINK1/Parkin-related mitophagy−restoring effects. In cell-based assays, glucocorticoid exposure suppressed, whereas GalR2 activation enhanced, mitochondrial membrane potential and PINK1/Parkin-related signaling.

**Conclusion:**

These findings identify a GalR2–mitochondrial axis in the ventral hippocampus that is disrupted by PS and associated with vulnerability to depression-like phenotypes. The complementary intra−vHPC infusion experiments establish a causal role for vHPC GalR2 signaling in rescuing mitochondrial deficits, directly demonstrating that intranasal AR−M1896 acts at least in part via the vHPC. This receptor–organelle pathway may represent a neurobiological mechanism linking early-life adversity to long-term affective dysfunction.

## 1 Introduction

PS is increasingly recognized as a major risk factor for depression and other stress-related psychiatric disorders in later life. Clinical studies have shown that maternal stress, anxiety, or depression during pregnancy increases the risk of emotional and behavioral problems, including depression and cognitive deficits, in the offspring (1–5). Consistently, rodent models of PS reproduce key features of this vulnerability, leading to depressive-like behaviors, impaired hippocampal-dependent learning and memory, and altered stress responsivity in the offspring (6–8). A converging body of evidence implicates long-lasting disruption of hypothalamic–pituitary–adrenal (HPA) axis homeostasis as a central mediator of these outcomes: PS offspring typically exhibit elevated basal and/or stress-induced glucocorticoid levels and impaired negative feedback, associated with region-specific alterations in hippocampal glucocorticoid receptor (GR) and mineralocorticoid receptor (MR) expression (9, 10). Chronic glucocorticoid excess and dysregulated GR/MR signaling are known to damage neuronal mitochondria, increase oxidative stress, and impair mitochondrial quality control (11, 12). In PS models, mitochondrial dysfunction and oxidative damage have been documented in the hippocampus, including decreased mitochondrial respiratory chain components, altered mitochondrial biogenesis, and increased markers of oxidative stress (13–15). Mitochondrial quality control relies critically on PINK1/Parkin-dependent mitophagy to identify and eliminate damaged organelles (16, 17), yet it remains unclear whether PS perturbs this mitophagy pathway in mood-regulating hippocampal circuits, particularly within the ventral hippocampus (vHPC), which plays a key role in emotional behavior and HPA axis feedback.

Galanin is expressed in several stress- and mood-related brain regions, including the locus coeruleus, dorsal raphe, and hippocampus (18, 19). It has also been implicated in the modulation of emotional behavior and affective disorders (20, 21). Among the three galanin receptors, GalR2 preferentially couples to Gq/11 and activates intracellular signaling cascades such as ERK and PI3K/Akt that support cell survival and neurotrophic processes (22). Pharmacological and genetic studies indicate that activation of GalR2 produces robust antidepressant- and anxiolytic-like effects, whereas blockade or reduced GalR2 signaling can have the opposite impact (23–25). Beyond its roles in the central nervous system, recent work has shown that galanin signaling preserves mitochondrial integrity and coordinates macrophage-associated fibro-inflammatory responses during myocardial infarction–reperfusion injury, directly linking galanin to the maintenance of mitochondrial homeostasis under stress (26). Together, these findings raise the possibility that, in the brain, GalR2 may act as a mitochondria-protective, stress-buffering mechanism that limits glucocorticoid-induced mitochondrial damage. However, it is unknown whether PS disrupts GalR2 signaling in the vHPC by impairing a GalR2-dependent PINK1/Parkin mitophagy axis, and how this might mechanistically link glucocorticoid excess to mitochondrial dysfunction and depressive-like behavior in the offspring.

In the present study, we used a well-established PS model to investigate how early-life glucocorticoid excess impacts the galanin system, mitochondrial quality control, and mood-related behaviors in offspring. We first tested the hypothesis that PS induces depressive-like behaviors in offspring, accompanied by elevated basal corticosterone levels and impaired glucocorticoid negative feedback in the vHPC, as reflected by reduced GR and altered expression of MR and Fkbp5. We then examined whether PS causes mitochondrial ultrastructural damage and downregulation of the PINK1/Parkin mitophagy machinery in the vHPC. To probe causality and downstream mechanisms, we modeled glucocorticoid excess *in vitro* by exposing GalR2-stably expressing HEK293 cells to high-dose corticosterone and assessed mitochondrial membrane potential as well as PINK1/Parkin expression. Finally, we evaluated whether pharmacological activation of GalR2 with the peptide agonist AR-M1896 could rescue glucocorticoid-induced mitochondrial damage, and whether intranasal administration of AR-M1896 ameliorates depressive-like behaviors in PS offspring in a GalR2-dependent manner. By delineating a glucocorticoid-sensitive GalR2–PINK1/Parkin mitophagy axis in the vHPC, our work aims to identify a novel mechanism by which PS programs long-lasting mitochondrial and behavioral vulnerability, and to provide provides initial support for that GalR2 activation—particularly via intranasal delivery—represents provides preliminary mechanistic insights for early-life stress–related mood disorders.

## 2 Materials and methods

### 2.1 Animals and PS model

All animals experiments were approved by the Animal Care Committee at Capital Medical University (Permit Number: AEEI-2025-706) and were carried out in accordance with National Institutes of Health Guide for the Care and Use of Laboratory Animals.

Adult male and female Sprague-Dawley (SD) rats (200–300 g) were used for breeding. Timed pregnancies were established by housing one male with two females overnight. The presence of a vaginal plug on the following morning was designated as gestational day 1 (GD1). Pregnant dams were then randomly assigned to either a control group or a PS group. Control dams remained undisturbed in their home cages throughout gestation. In contrast, Pregnant dams in the PS group were subjected to restraint stress from gestational day (GD) 14 to GD21. Restraint was performed using a commercially available rat restrainer (Z-350 restraint tube; polycarbonate, PC; external dimensions: length 200 mm, outer diameter 78 mm, inner diameter 70 mm), which restricted body movement while allowing adequate ventilation. During restraint, nose remained exposed. Dams underwent three restraint sessions per day, each lasting 4 min. To reduce predictability and minimize habituation/associative learning to fixed time cues, the start time of each session was varied randomly from day to day. All offspring were weaned on postnatal day 21 (PND21), and only male offspring were used for subsequent experiments to avoid potential confounding effects of the estrous cycle. Behavioral tests were conducted when the offspring reached adulthood at two months of age.

### 2.2 Behavioral tests

All behavioral tests were conducted during the light phase (between 4:00 PM and 22:00 PM) by an experimenter blind to the treatment groups.

#### 2.2.1 Sucrose preference test

Anhedonia-like behavior was evaluated over a 48 h protocol. Rat were first habituated to two bottles of 1% sucrose for 12 h, followed by a 12 h period with one sucrose and one water bottle (with positions reversed after 6 h). After a 12 h water deprivation period, Rat were placed in a novel cage for a 30 min acclimation and then presented with pre-weighed bottles containing 1% sucrose and water for 1 h. Sucrose preference was calculated as [sucrose intake/(sucrose intake + water intake)× 100%.

#### 2.2.2 Forced swim test

We measured despair-like behavior in rats using the forced swim test (27).The test was conducted after 4:00 PM. Rats were placed individually into a transparent cylindrical tank (height: 100 cm, diameter: 60 cm) filled with water (25 ± 1 °C) to a depth of 75 cm. Their behavior was recorded for 6 minutes by a camera positioned above the tank. The duration of immobility (defined as making only minimal movements necessary to keep the head above water) during the last 4 minutes was scored by an observer blinded to the experimental conditions, or quantified using ANY-maze video tracking software.

### 2.3 Measurement of plasma corticosterone levels

Plasma corticosterone (CORT) levels were measured using an enzyme-linked immunosorbent assay (ELISA). To minimize circadian variation, blood samples were collected before 9:00 a.m. Blood was obtained by tail-tip sampling, collected into anticoagulant tubes, and centrifuged at 4 °C (3,000 rpm, 10 min) to separate plasma. Plasma samples were stored at −80 °C until analysis. CORT concentrations were determined using a commercial Rat CORT ELISA kit (JYM0590Ra; Jiyinmei, Wuhan, China) according to the manufacturer’s instructions. Absorbance was measured at 450 nm, and concentrations were calculated from a standard curve with appropriate dilution correction.

### 2.4 Drug administration

AR-M1896 (Qiangyao Biotechnology, Shanghai, China), a selective GALR2 agonist, was dissolved in normal saline containing 5% α-cyclodextrin. Based on previous intranasal studies of galanin-related peptides and the higher *in vitro* potency of AR-M1896, a dose of 20 nmol/rat (1 mM, total volume 20 μL/rat, 10 μL per nostril) was selected. AR-M1896 was administered intranasally once daily for 7 consecutive days under brief isoflurane anesthesia. Behavioral tests were performed 24 h after the final administration. Vehicle-treated controls received the same volume of Vehicle (saline with 5% α-cyclodextrin) under identical anesthesia and handling procedures.

#### 2.4.1 Surgical procedures and drug administration

Adult male rats were anesthetized using an isoflurane vaporizer (RWD Life Science, Shenzhen, China) with an oxygen flow rate of 0.5 L/min. Anesthesia was induced with 4% isoflurane in an induction chamber and maintained with 1% isoflurane delivered via a nose cone mounted on a stereotaxic frame. The rats were then placed in the stereotaxic frame (RWD Life Science and Technology). Bilateral guide cannulas (RWD Life Science and Technology) were implanted into the ventral hippocampus (VH) at the following coordinates relative to bregma: ML ± 4.8 mm, AP -5 mm, DV -7.8 mm (23). The cannulas were secured with dental cement and stainless steel screws and sealed with stylets. After surgery, rats received a 7-day recovery period.

For drug administration, AR-M1896 (Qiangyao Biotechnology, Shanghai, China) was dissolved in 0.9% saline to a concentration of 1 mM (1 nmol/μL) (28). Each rat received a unilateral intra-VH infusion of 1 μL of AR-M1896 once daily for 7 consecutive days, while the contralateral vHPC received an equal volume of 0.9% saline (Vehicle) under the same schedule. Infusions (1 μL per side) were administered over 5 minutes, and the injection needle was left in place for 20 s after each infusion to ensure complete drug delivery. The dose and regimen were selected based on previous studies demonstrating therapeutic effects. Behavioral tests were conducted 24 h after the last drug administration.

### 2.5 Measurement of ATP Levels in the Ventral Hippocampus

ATP levels in the vHPC were quantified using a luciferase-based bioluminescence assay (Enhanced ATP Assay Kit, S0027, Beyotime, Shanghai, China). Frozen vHPC tissues were homogenized, centrifuged, and ATP content was measured according to the manufacturer’s protocol. Luminescence was normalized to total protein content determined by a BCA assay.

### 2.6 Cell culture and treatments

Human embryonic kidney 293T (HEK293T) cells and HEK293 cells stably expressing the galanin receptor 2 (GalR2) (29) were cultured in Dulbecco’s Modified Eagle Medium (DMEM) supplemented with 10% fetal bovine serum (FBS) and 1% penicillin-streptomycin. Cells were maintained in a humidified incubator at 37 °C with 5% CO_2_.

For transfection experiments, Lipofectamine 3000 reagent (L3000008, Thermo Fisher Scientific, MA, USA) was used according to the manufacturer’s instructions. For pharmacological treatments, cells were exposed to varying concentrations of corticosterone (HY-B1618, CORT; MedChemExpress, MCE, NJ, USA) to mimic glucocorticoid excess. To assess the effect of GalR2 activation, the selective GalR2 agonist AR-M1896 (Qiangyao Biotechnology, Shanghai, China) was co-administered with CORT.

### 2.7 Mitochondrial membrane potential and morphology assessment

Mitochondrial membrane potential (ΔΨm) was assessed using the JC-1 dye (C2003, Beyotime Biotechnology, Shanghai, China). Cells were incubated with JC-1 working solution in a dilution buffer at 37 °C for 15 minutes, followed by two washes with ice-cold dilution buffer. Fluorescence intensity was quantified using a high-content imaging system (Thermo Scientific™ CellInsight™ High-Content Screening Platform, MA, USA). The JC-1 assay was performed in four independent experiments (n = 4 biological replicates). Within each well, nine microscopic fields were imaged, and fluorescence measurements from these fields were averaged to generate one value per well. Statistical analysis was then performed using the well/independent experiment average as the statistical unit, thereby avoiding inflation of sample size through field-level pseudo−replication.

For mitochondrial morphology analysis, cells were stained with MitoTracker Red FM (M22425, Thermo FisDher Scientific, MA, USA) at a final concentration of 100 nM for 20 minutes at 37 °C. After two washes with phosphate-buffered saline (PBS), fresh culture medium was added, and mitochondrial morphology images were acquired in standard confocal mode (Leica TCS SP8 STED, Wetzlar, Germany). Acquired images were analyzed using the Mitochondrial Network Analysis (MiNA) toolset in Fiji/ImageJ to quantify parameters including mean mitochondrial area, perimeter, and form factor.

### 2.8 Molecular biology assays

Total RNA was extracted from vHPC tissue or cultured cells using TRIzol reagent (15596026CN, Invitrogen, CA, USA) and reverse-transcribed into cDNA using a PrimeScript RT reagent kit (AG11701, Accurate Biology, Changsha, China). Quantitative real-time PCR (qPCR) was performed on a CFX96 Touch Real-Time PCR Detection System (Bio-Rad) with SYBR Green Master Mix (AG11728, Accurate Biology, Changsha, China). Gene-specific primers for target genes (Fkbp5, Mr) and housekeeping genes (GAPDH) were designed and validated for efficiency (see **Table 1**).

**Table 1:** Primer sequences used for quantitative real-time PCR.

Gene	Forward primer (5′→3′)	Reverse primer (5′→3′)
MR	CAGCTCACCTCCATTACGCA	CTTCACGACCTGGCTCATCT
FKBP5	TTCCCTCGAACGCAACTCTC	GTCGTGGTCTTCTCCTTCGC
GAPDH	TGGGTGTGAACCATGAGAAG	GAGTCCTTCCACGATACCAAAG

For Western blot analysis, total protein was extracted from vHPC tissue or cells using RIPA lysis buffer containing protease and phosphatase inhibitors. Protein concentration was determined using a BCA Protein Assay Kit (23225, Thermo Fisher Scientific, MA, USA). Equal amounts of protein were separated by SDS-PAGE and transferred onto polyvinylidene difluoride (PVDF) membranes. The membranes were blocked with 5% non-fat milk in Tris-buffered saline with Tween 20 (TBST) for 1 hour at room temperature and then incubated overnight at 4 °C with primary antibodies against GAPDH (1:5000, 60004-1-Ig, Proteintech, USA), GR (1:1000, 12041S, Cell Signaling Technology, MA, USA), GalR2 (1:1000, YT1845, Immunoway, TX, USA), Parkin (1:1000, R381626, Zenbio, Chengdu, China), and PINK1 (1:1000, 507131, Zenbio, Chengdu, China). After washing, the membranes were incubated with secondary antibodies (1:10000, 925–68021 and 926-32210, 1:10,000, LI-COR, NE, USA) for 1 hour at room temperature. Protein bands were visualized and quantified using an Odyssey CLx Imaging System (LI-COR Biosciences).

### 2.9 Transmission Electron Microscopy (TEM)

For mitochondrial ultrastructural analysis, rats were deeply anesthetized and transcardially perfused with saline followed by 4% paraformaldehyde. The ventral hippocampus (vHPC) was dissected, post−fixed in 2.5% glutaraldehyde, and processed for TEM. Samples were post−fixed with osmium tetroxide, dehydrated, embedded in Epon resin, and sectioned. Sections were stained with uranyl acetate and lead citrate and examined under a transmission electron microscope (Hitachi HT7800, Tokyo, Japan). For quantitative assessment, three rats per group (Control and PS) were used. From each animal, three vHPC sections were examined, and at least 50 mitochondria per section were evaluated by an investigator blinded to group assignment.

### 2.10 Statistical analysis

Data are presented as mean ± SEM. Statistical analyses were performed using GraphPad Prism 9.0. Comparisons between two groups were conducted using an unpaired two-tailed Student’s t-test. Comparisons among multiple groups were performed using one-way ANOVA with homogeneity of variances verified by Brown−Forsythe test, followed by Tukey’s HSD *post hoc* test. A value of *P*<0.05 was considered statistically significant.

## 3 Results

### 3.1 PS induces depressive-like behaviors and HPA axis hyperactivity, which are rescued by GalR2 activation via intranasal and intra-vHPC routes

**Figure 1:**
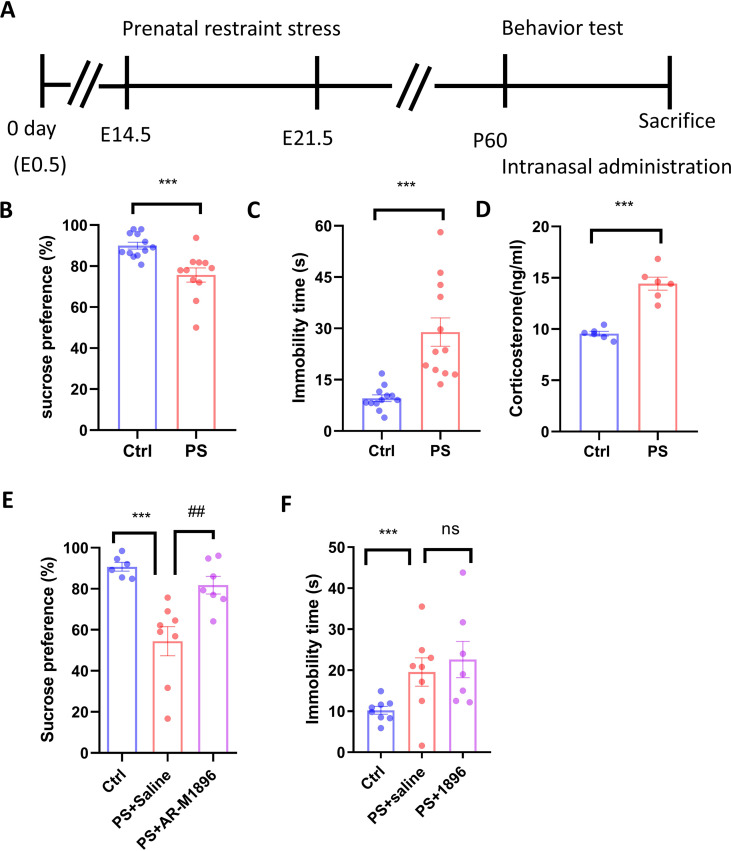
PS induces depressive like behaviors and HPA axis hyperactivity, which are rescued by GalR2 activation via intranasal and intra vHPC routes. **(A)** Experimental timeline illustrating PS exposure (GD14–GD21), followed by behavioral assessments in adult offspring at postnatal day 60. The GalR2 agonist AR-M1896 was delivered intranasally 24 h before behavioral testing. Intranasal (i.n.) Intra ventral hippocampal (i.v.). **(B–D)** Validation of the PS model showing decreased sucrose preference, increased immobility time in the forced swim test, and elevated plasma corticosterone levels in PS offspring compared with controls. Sample sizes were as follows: **(B)** Ctrl, n = 12; PS, n = 12. **(C)** Ctrl, n = 12; PS, n = 12. **(D)** Ctrl, n = 6; PS, n = 6. **(E, F)** AR-M1896 restored sucrose preference, whereas its effect on immobility time did not reach statistical significance and showed a non-significant trend toward reduced immobility, (Ctrl, n = 8; PS + saline, n = 7; PS + AR-M1896, n = 8). Data are presented as mean ± SEM. Statistical analysis was performed using an unpaired two-tailed Student’s t-test for **(B–D)** and three−group comparisons were performed using one−way ANOVA, with homogeneity of variances verified by Brown−Forsythe test (*P*  >  0.05), followed by Tukey’s HSD *post hoc* test for**(E, F)**. ***P < 0.001 vs. Ctrl; *P* < 0.01 vs. PS + saline.

As depicted in the figure, prenatal restraint stress (PS) was applied from GD14 to GD21. At postnatal day 60 (P60), PS offspring received intranasal (i.n.) or intra-ventral hippocampal (i.v.) infusion of the GalR2-selective agonist AR-M1896 for one week, after which depressive-like behaviors were assessed to evaluate the behavioral effects of PS and the potential antidepressant efficacy of GalR2 activation. The experimental procedure is outlined in **Figure 1A**.

As shown in **Figures 1B–D**, PS offspring exhibited pronounced depressive‐like phenotypes characterized by a significant reduction in sucrose preference (***P  <  0.001), increased immobility time in the forced swim test (***P  <  0.001), and elevated plasma corticosterone levels (***P  <  0.001) compared with control animals. These findings confirm long−lasting behavioral and endocrine consequences of PS exposure, consistent with previous reports of HPA axis dysregulation and affective deficits in PS models.

Importantly, intranasal administration of AR−M1896 effectively reversed the anhedonic phenotype induced by PS (**Figure 1E**), restoring sucrose preference to near−control levels (ns *P* > 0.05 vs. Ctrl). Although AR−M1896 treatment produced a trend toward reduced immobility time in the forced swim test, the difference did not reach statistical significance (**Figure 1F**). Together, these data indicate that GalR2 activation via an intranasal route alleviates PS−induced behavioral deficits, particularly hedonic impairment, and exerts partial antidepressant−like effects.

Overall, the results demonstrate that PS results in persistent depression−like behaviors and hypercorticosteronemia in adult offspring, while activation of GalR2 through intranasal delivery of AR−M1896 shows efficacy in mitigating stress−related affective symptoms, particularly anhedonia.

### 3.2 PS disrupts glucocorticoid signaling and downregulates GalR2 expression in the ventral hippocampus

**Figure 2:**
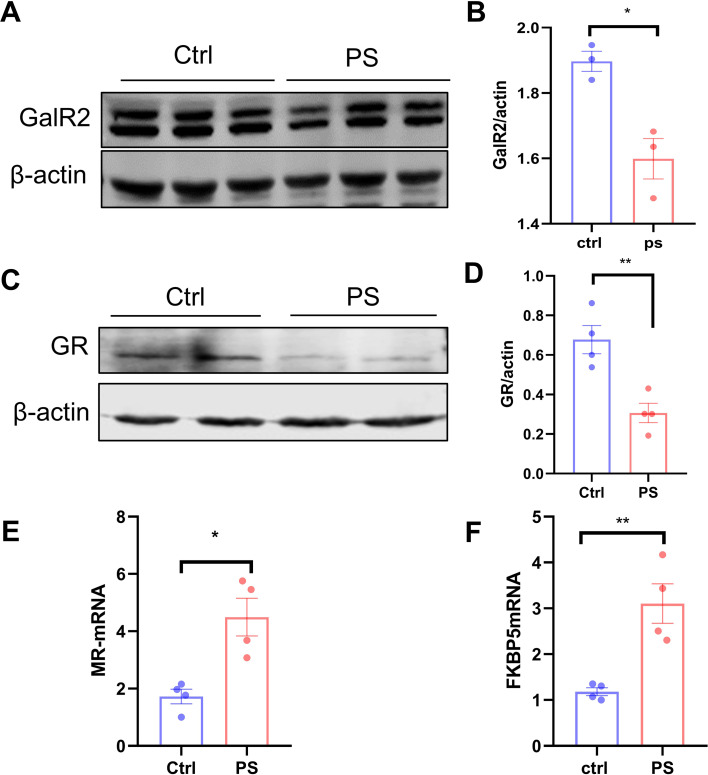
PS disrupts glucocorticoid signaling and downregulates GalR2 expression in the ventral hippocampus. **(A, B)** Representative immunoblot images and quantification showing reduced GR protein levels in the vHPC of PS offspring. Ctrl, n = 6; PS, n = 6. **(C, D)** Quantitative real-time PCR analysis showing increased MR and Fkbp5 mRNA expression in the vHPC of PS offspring. Ctrl, n = 6; PS, n = 6. **(E, F)** Representative immunoblot images and quantification showing decreased GalR2 protein expression in the vHPC of PS offspring. Ctrl, n = 6; PS, n = 6. Data indicate impaired glucocorticoid feedback and reduced galanin receptor signaling in PS vHPC. Data are presented as mean ± SEM. Statistical analysis was performed using an unpaired two-tailed Student’s t-test. *P < 0.05,**P < 0.01 vs. Ctrl.

Given the pronounced behavioral and endocrine disturbances observed in PS offspring, we next examined key components of glucocorticoid signaling and galanin receptor expression within the ventral hippocampus (vHPC), a region critically involved in stress regulation and affective control (**Figure 2**).

Western blot analysis revealed that glucocorticoid receptor (GR) protein levels were significantly reduced in the vHPC of PS rats compared with age−matched controls (n  =  6 per group, **P <0.01; **Figures 2A,B**). In contrast, quantitative real−time PCR demonstrated significant elevations in mineralocorticoid receptor (MR) and FK506−binding protein 5 (Fkbp5) mRNA expression (n = 6 per group, *P <  0.05 and **P <0.01, respectively; **Figures 2C,D**). This reciprocal shift—lower GR protein with increased MR and Fkbp5 transcripts—indicates disrupted glucocorticoid negative feedback and heightened tissue sensitivity to stress hormones.

Importantly, we found that GalR2 protein expression in the vHPC was markedly decreased in PS offspring relative to controls (n  =  6 per group, *P < 0.05; **Figures 2E,F**). Because GalR2 exerts neuroprotective and antidepressant actions in hippocampal circuits, its downregulation suggests that PS compromises galanin−mediated signaling in mood−relevant hippocampal pathways.

Together, these findings demonstrate that PS induces persistent molecular changes in the vHPC characterized by impaired glucocorticoid feedback and diminished GalR2 expression, potentially contributing to HPA axis hyperactivity and depressive−like behavior observed in adulthood.

### 3.3 PS impairs mitochondrial bioenergetics and PINK1/Parkin signaling in the ventral hippocampus, and AR-M1896 treatment rescues these deficits

**Figure 3:**
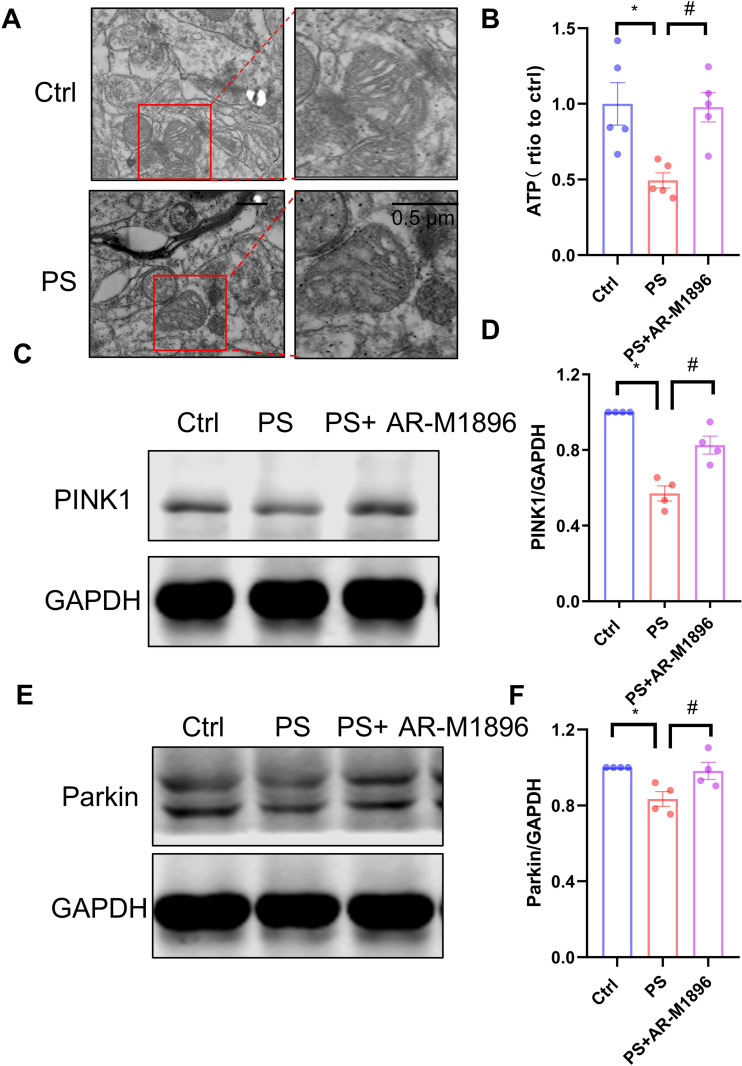
PS impairs mitochondrial bioenergetics and PINK1/Parkin signaling in the ventral hippocampus, and AR-M1896 treatment rescues these deficits. **(A)** Transmission electron microscopy (TEM) images showing mitochondrial ultrastructure in control and PS rats. Mitochondria in controls displayed elongated morphology with dense cristae, while PS mitochondria were swollen, fragmented, and exhibited disrupted or vacuolated cristae, Scale bar  =  0.5 μm. **(B)** ATP levels in the vHPC were significantly reduced in PS offspring compared with controls, Intranasal administration of AR−M1896 restored ATP levels to near−control values (n = 5 per group, *P <  0.05  vs. Ctrl, #*P*<0.05 vs. PS). **(C–F)** Western blot analysis of PINK1 and Parkin showing downregulated protein expression in the vHPC of PS offspring, and significant upregulation following AR−M1896 treatment(n = 4 per group, *P  <  0.05  vs. Ctrl, #*P*<0.05 vs. PS). Data are expressed as mean  ±  SEM. Three−group comparisons were performed using one−way ANOVA, with homogeneity of variances verified by Brown−Forsythe test, followed by Tukey’s HSD *post hoc* test.

To determine whether PS alters mitochondrial function and quality control in the ventral hippocampus (vHPC), we quantified ATP levels, examined mitochondrial ultrastructure by transmission electron microscopy (TEM), and assessed the expression of PINK1 and Parkin, two key upstream regulators of mitochondrial quality control(**Figure 3**).

Biochemical analysis revealed a significant reduction in ATP content in the vHPC of PS offspring compared with controls (*P  < 0.05 vs. Ctrl; **Figure 3B**), Notably, intranasal administration of AR-M1896 restored ATP levels to near-control values, which were significantly higher than those in the PS group(**Figure 3B**). Consistent with this finding, TEM analysis of vHPC neurons demonstrated marked mitochondrial ultrastructural abnormalities in PS rats. Compared with the elongated mitochondria with densely packed and intact cristae observed in control animals, mitochondria from PS offspring appeared swollen and fragmented, exhibiting disorganized or vacuolated cristae (**Figure 3A**).

Western blot analysis further confirmed a disruption of the PINK1/Parkin−dependent quality control mechanism in PS offspring, showing significant downregulation of both PINK1 and Parkin protein levels in the vHPC relative to controls. Treatment with AR-M1896 significantly upregulated PINK1 and Parkin expression compared with the PS group (*P<0.05 vs. Ctrl, #*P*  <  0.05 vs. PS; **Figures 3C–F**), indicating restoration of this signaling pathway.

Together, these results demonstrate that PS induces mitochondrial dysfunction in the vHPC, characterized by reduced ATP production, structural damage, and downregulation of the PINK1/Parkin pathway. Intranasal delivery of the GalR2 agonist AR-M1896 effectively reverses the PS-induced deficits in ATP levels and PINK1/Parkin expression, supporting the role of GalR2-mediated mitochondrial homeostasis in the vHPC.

**Figure 4:**
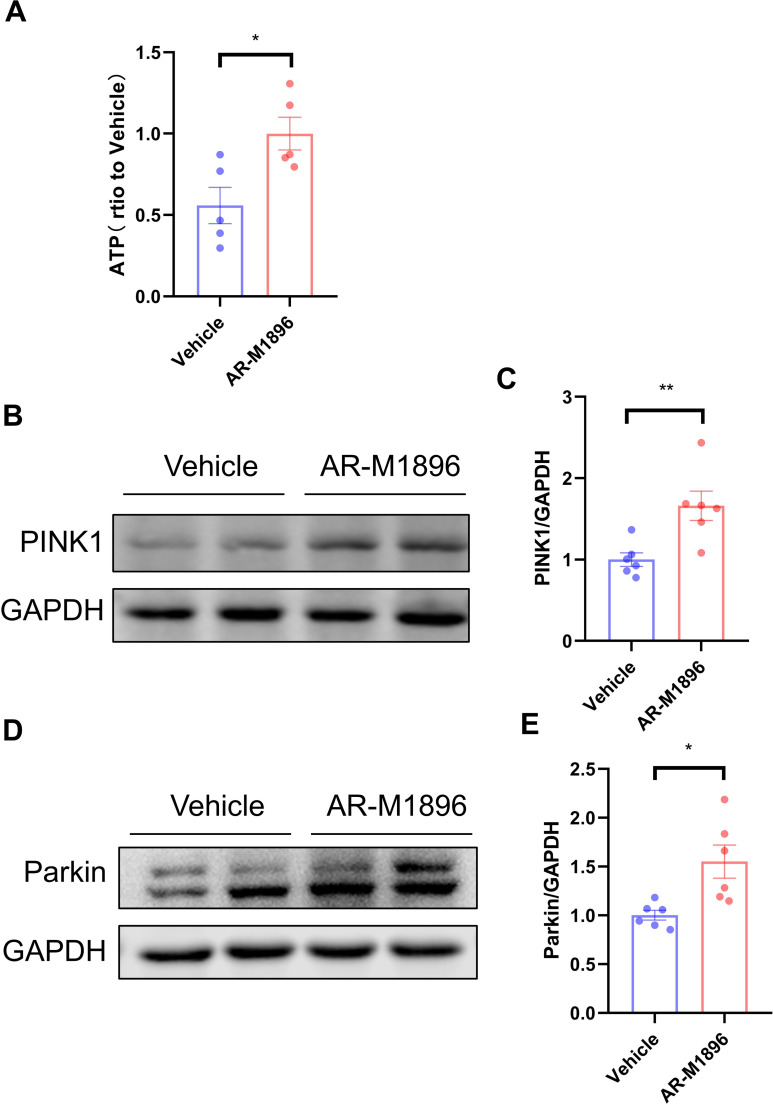
Direct intra−vHPC infusion of AR−M1896 recapitulates the rescue of ATP, PINK1, and Parkin levels in PS rats. **(A)** ATP levels in the vHPC of PS rats following unilateral infusion of AR−M1896 (1 mM, 1 μL/day for 7 days) compared with the contralateral Vehicle (0.9% saline) side. AR−M1896 significantly elevated ATP content relative to Vehicle control (n  =  6 per group, *P  <  0.01 vs. Vehicle). **(B)** Representative Western blots showing PINK1 protein expression in the vHPC of Vehicle and AR−M1896−treated sides. **(C)** Densitometric quantification of PINK1 normalized to GAPDH. AR−M1896 treatment markedly increased PINK1 levels compared with Vehicle (n  =  6 per group, **P  <  0.001 vs. Vehicle). **(D)** Representative Western blots showing Parkin protein expression. **(E)** Densitometric quantification of Parkin normalized to GAPDH. AR−M1896 significantly upregulated Parkin expression compared with the Vehicle side (n  =  6 per group, *P  <  0.01 vs. Vehicle). Data are presented as mean ± SEM. Comparisons between the two brain regions (Vehicle vs. AR‑M1896) from the same animals were performed using paired two‑tailed Student’s t‑test. (*P* > 0.05).

To directly test whether the effects of intranasal AR−M1896 are mediated specifically by the vHPC, we performed unilateral intra−vHPC cannulation in PS rats and infused AR−M1896 (1 mM, 1  μL/day for 7 days) on one side while the contralateral side received Vehicle (0.9% saline). As shown in **Figure 4**, AR−M1896 infusion significantly elevated ATP levels in the ipsilateral vHPC compared with the Vehicle−treated side(**Figure 4A**). Western blot analysis of vHPC tissues revealed that AR−M1896 treatment markedly increased the protein expression of PINK1 (**Figures 4B,C**) and Parkin (**Figures 4D,E**) relative to Vehicle controls. These results demonstrate that direct activation of GalR2 within the vHPC recapitulates the protective effects of intranasal AR−M1896 on mitochondrial bioenergetics and PINK1/Parkin−mediated mitophagy. Together, these findings establish that intranasal AR−M1896 acts at least in part through the vHPC to reverse PS−induced mitochondrial deficits.

### 3.4 GalR2 activation protects HEK293-GalR2 cells from corticosterone-induced mitochondrial dysfunction and fragmentation

**Figure 5:**
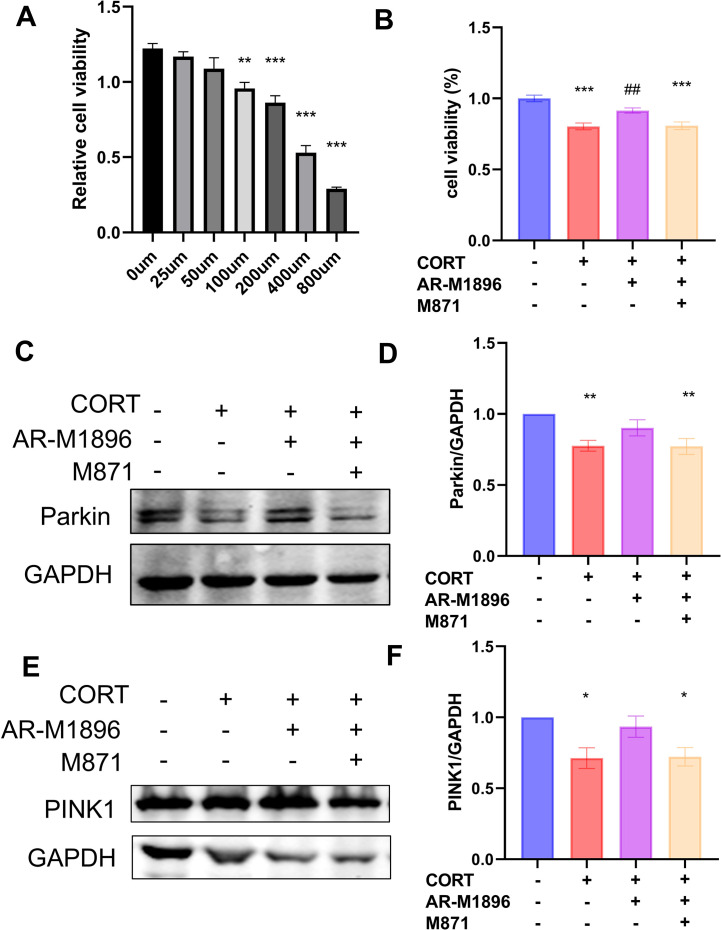
GalR2 activation alleviates corticosterone-induced cytotoxicity and restores PINK1/Parkin expression in HEK293-GalR2 cells. **(A)** CORT treatment (10–800  µM, 24 h) reduced cell viability in a dose-dependent manner, with maximal reduction observed at 100  µM (n = 3, **P  <  0.01, ***P  <  0.001 vs. Vehicle). **(B)** AR-M1896 (0.1  µM) alone had no effect on viability but significantly prevented the cytotoxicity induced by 100  µM CORT (n = 3, ##*P*  <  0.01 vs. CORT alone). Co-application of M871 (0.1  µM) abolished this protective effect (n = 3, ***P  <  0.001 vs. Vehicle). **(C–F)** Western blot analysis showing that CORT suppresses PINK1 and Parkin expression, while AR-M1896 restores both proteins to near-control levels; M871 co-treatment negated these restorative effects (n = 6, *P  <  0.05, **P  <  0.01 vs. Vehicle). n indicates the number of independent experiments. Data are presented as mean  ±  SEM, analyzed by one-way ANOVA with homogeneity of variances verified by Brown−Forsythe test, followed by Tukey’s HSD *post hoc* test. (*P*  <  0.05).

To delineate the mechanisms by which GalR2 activation modulates glucocorticoid-related mitochondrial injury, HEK293 cells stably expressing GalR2 (HEK293-GalR2) were exposed to increasing concentrations of corticosterone (CORT; 10–800 µM) for 24 h. CORT decreased cell viability in a dose-dependent manner, with maximal toxicity at 100 µM (n = 3 from three independent experiments, ***P < 0.001 vs. Vehicle; **Figure 5A**). Treatment with the GalR2-preferring agonist AR-M1896 (0.1 µM) alone did not affect viability, but significantly ameliorated the decline induced by 100  µM CORT (^##^*P* < 0.01 vs. CORT alone). Co-application of the GalR2 antagonist M871 (0.1 µM) abolished the protective effect, restoring cytotoxicity to levels comparable to CORT treatment (***P < 0.001 vs. Vehicle; **Figure 5B**).

Consistent with *in vivo* findings, exposure to 100  µM CORT for 24 h markedly reduced PINK1 and Parkin protein expression in HEK293-GalR2 cells (*P < 0.05 and **P < 0.01 vs. Vehicle; **Figures 5C–F**). Co-treatment with AR-M1896 effectively restored PINK1 and Parkin levels toward control values (*P* > 0.05 vs. Vehicle), whereas M871 co-administration prevented this recovery (*P < 0.05 and **P < 0.01 vs. Vehicle). These data indicate that GalR2 activation is both necessary and sufficient to maintain PINK1/Parkin-dependent mitophagy under glucocorticoid stress.

**Figure 6:**
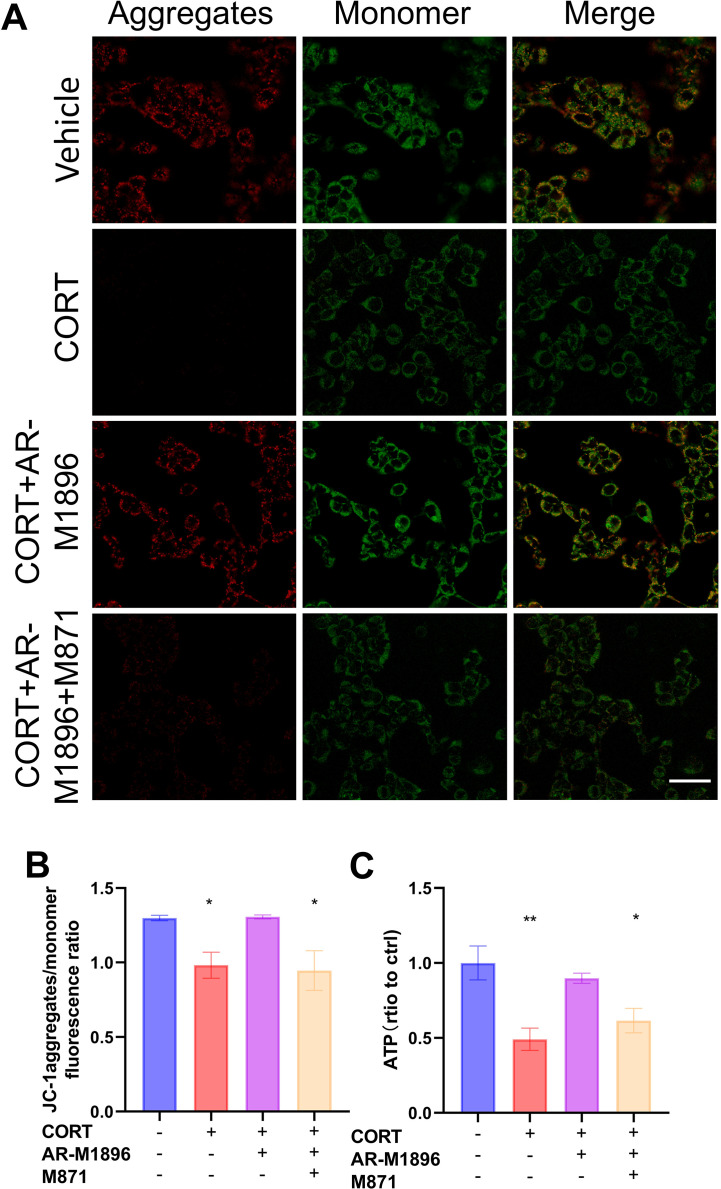
GalR2 activation preserves mitochondrial membrane potential and ATP production under glucocorticoid stress. **(A, B)** JC-1 staining showing reduced mitochondrial membrane potential after 100 µM CORT exposure (n = 4, *P  <  0.05 vs. Vehicle), which was significantly rescued by AR-M1896 (n = 4, *P*  >  0.05 vs. Vehicle). Co-treatment with M871 abrogated this effect. **(C)** ATP levels were significantly decreased after CORT treatment compared with the Vehicle group (n = 4, **P  <  0.05 vs. Vehicle). Treatment with AR-M1896 significantly rescued the CORT-induced reduction in ATP, while the addition of the GalR2 antagonist M871 partially blocked this effect (n = 4, *P*  >  0.05 vs. Vehicle). Data are expressed as ratios relative to the Vehicle group. Data are presented as mean  ±  SEM from four independent experiments, analyzed by one-way ANOVA with homogeneity of variances verified by Brown−Forsythe test, followed by Tukey’s HSD *post hoc* test. (*P*  <  0.05). Scale bar  =  50 μm.

**Figure 7:**
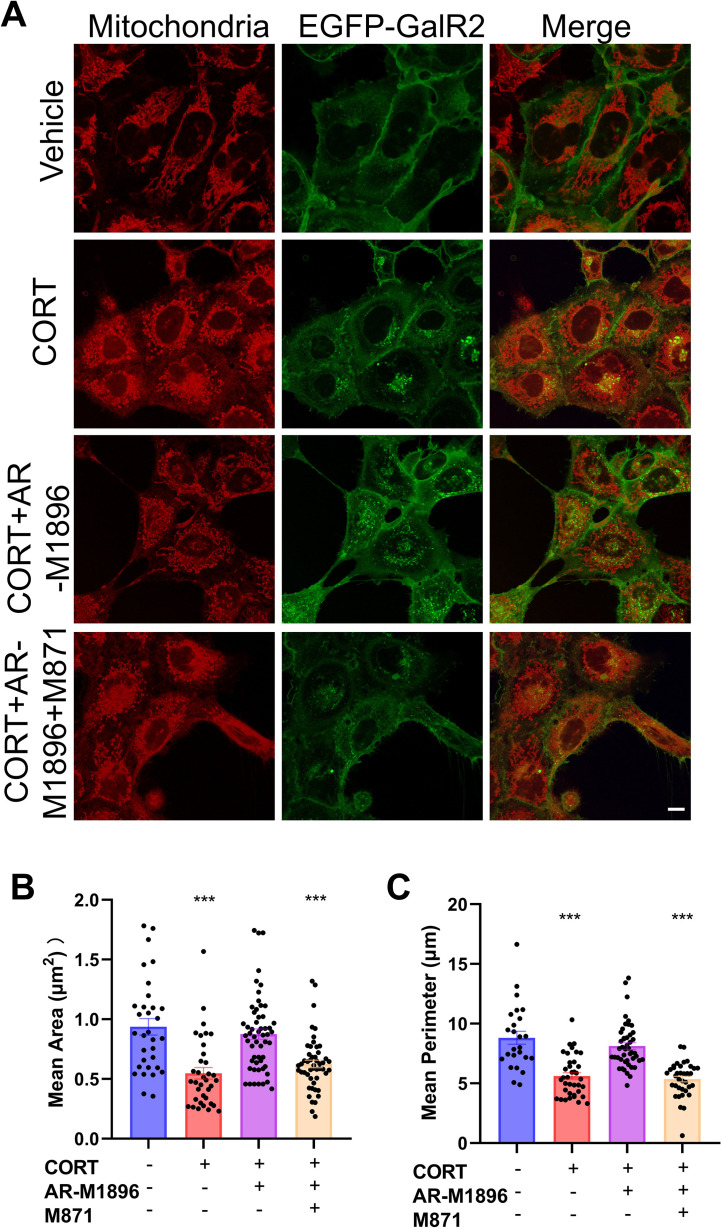
GalR2 activation preserves mitochondrial morphology under glucocorticoid stress. **(A–C)** Confocal and morphometric analyses showing mitochondrial fragmentation induced by CORT (decreased area and perimeter; ***P  <  0.001 vs. Vehicle). AR-M1896 restored elongated mitochondrial morphology (*P*  > 0.05 vs. Vehicle), while M871 co-administration abolished the rescue (***P  <  0.001 vs. Vehicle). Data demonstrate GalR2-dependent protection against CORT-induced mitochondrial dysfunction. n ≥ 35 cells from three independent experiments, with at least 11 cells analyzed per experiment. Data are presented as mean  ±  SEM, analyzed by one-way ANOVA with homogeneity of variances verified by Brown−Forsythe test, followed by Tukey’s HSD *post hoc* test. (*P*  <  0.05). Scale bar  =  10 μm.

To further assess mitochondrial function and morphology, we examined the effects of GalR2 activation on CORT-induced mitochondrial depolarization and structural damage (**Figure 6**). JC-1 staining revealed that 100 µM CORT significantly decreased mitochondrial membrane potential compared with Vehicle (*P  < 0.05 vs. Vehicle), while AR-M1896 treatment preserved membrane potential (*P* > 0.05 vs. Vehicle). The protective effect was nullified by M871 co-application, confirming a GalR2-dependent mechanism (**Figures 6A,B**). In parallel, intracellular ATP levels were significantly decreased by CORT treatment, while AR-M1896 attenuated this reduction; the effect was partially reversed by M871 co-application (**Figure 6C****).**Morphometric analysis demonstrated that CORT exposure led to pronounced mitochondrial fragmentation, with reduced average area and perimeter (**P < 0.001 vs. Vehicle; **Figures 7A–C**). AR-M1896 markedly restored mitochondrial morphology toward elongated forms (*P* > 0.05 vs. Vehicle), whereas M871 again abolished this rescue.

Together, these findings demonstrate that activation of GalR2 enhances cellular resilience to glucocorticoid toxicity by preserving PINK1/Parkin-dependent mitophagy, maintaining mitochondrial membrane potential, and preventing structural fragmentation.

## 4 Discussion

The present study identifies concurrent alterations in a glucocorticoid-sensitive GalR2–PINK1/Parkin-associated mitochondrial regulatory axis that may together contribute to long−lasting depressive−like behavior in offspring following prenatal stress. We show that PS induces anhedonia and behavioral despair, accompanied by elevated basal corticosterone and altered glucocorticoid signaling in the vHPC, including reduced GR protein and increased MR and Fkbp5 expression and decreased GalR2 expression. Intranasal administration of the GalR2 agonist AR−M1896 restored sucrose preference in PS offspring. At the cellular level, PS reduces ATP content, causes pronounced mitochondrial ultrastructural damage and downregulates the mitophagy regulators PINK1 and Parkin in the vHPC. Intranasal AR−M1896 also restored ATP levels and the protein expression of PINK1 and Parkin in the vHPC. To further demonstrate that AR−M1896 acts within the hippocampus, we performed direct intra−vHPC infusion of AR−M1896, which similarly restored ATP levels and PINK1/Parkin expression. *In vitro*, high−dose corticosterone recapitulated these mitochondrial deficits in GalR2−expressing cells, and AR−M1896 rescued them in a GalR2−dependent manner. Together, these findings support a model in which PS−induced glucocorticoid excess and GalR2 downregulation converge to impair PINK1/Parkin−dependent mitophagy and mitochondrial integrity in the vHPC including reduced ATP production, thereby contributing to depressive phenotypes that can be reversed by GalR2 activation.

HPA axis hyperactivity and impaired glucocorticoid feedback are hallmark biological features of major depression (30, 31). The hippocampus, particularly its ventral subdivision, plays a crucial role in negative feedback regulation of the HPA axis and in the control of emotional behavior (32, 33). PS offspring exhibit elevated basal corticosterone and a shift in hippocampal glucocorticoid signaling. This shift in the vHPC was characterized by reduced GR protein and increased MR and Fkbp5 mRNA. Decreases in hippocampal GR and/or altered GR: MR balance have been linked to exaggerated stress responses and depressive−like behavior in rodents (6, 34–36), while increased FKBP5, a co−chaperone that reduces GR sensitivity, has been associated with stress−related psychiatric disorders in humans (37–39). Our data therefore support the notion that PS programs enduring HPA axis dysregulation via region−specific alterations in hippocampal glucocorticoid signaling, with the vHPC representing a key node for mood−related outcomes.

In addition to HPA axis perturbations, our results demonstrate that PS induces substantial mitochondrial pathology in the vHPC. Electron microscopy revealed swollen, fragmented mitochondria with disrupted cristae, accompanied by reduced ATP content and decreased PINK1 and Parkin protein levels. Mitochondrial dysfunction and oxidative stress in the hippocampus, characterized by decreased respiratory chain components, reduced mitochondrial DNA copy number, and increased oxidative damage, have been documented in PS models (11–13, 15). The current findings extend these observations by implicating defective PINK1/Parkin−dependent mitochondrial quality control pathway, as a mechanistic substrate for PS−induced mitochondrial impairment in mood−relevant vHPC circuits. Disruption of this pathway leads to accumulation of dysfunctional organelles and oxidative stress (16, 17). Thus, mitochondrial quality control may play a critical role in the long-term mood consequences of early-life stress.

A major novel aspect of this study is the identification of GalR2 as a potential upstream regulator of this mitochondrial vulnerability in the vHPC. Galanin and its receptors are known to be involved in stress and mood regulation (20, 21, 40). Notably GalR2 preferentially couples to Gq/11, mediating neurotrophic and anti−apoptotic effects, whereas GalR1/GalR3 couple to Gi/o (22). Pharmacological studies show that GalR2 activation produces antidepressant effects, while GalR1/GalR3 activation has pro−depressive actions (24, 40). Concurrently, PS offspring exhibited marked mitochondrial structural damage, reduced ATP production, and decreased expression of PINK1 and Parkin - key molecules in the mitochondrial quality control pathway - in the vHPC.

Given the emerging evidence that GalR2 signaling can influence mitochondrial function, we asked whether the observed reduction in GalR2 contributes to the mitochondrial deficits in the vHPC. To address this question, we performed both intranasal and direct intra−vHPC administration of the GalR2−selective agonist AR−M1896, and examined whether restoring GalR2 activity could rescue mitochondrial abnormalities and depressive−like behaviors.

To further determine whether the observed mitochondrial impairments are causally related to reduced GalR2 signaling, we performed a complementary experiment in a separate cohort of PS offspring using unilateral intra−vHPC infusion of the GalR2−selective agonist AR−M1896 (with contralateral Vehicle as control). After one week of local vHPC delivery, we found that direct GalR2 activation significantly increased ATP levels and upregulated PINK1 and Parkin expression specifically in the infused vHPC (**Figure 4**), indicating that GalR2 activation within the vHPC is sufficient to rescue PS−induced mitochondrial dysfunction.

Because intra−vHPC infusion is technically invasive and surgically demanding, we also explored intranasal administration as a less invasive translational approach. Previous studies have demonstrated that intranasal delivery of peptide drugs enables rapid transport to the brain via olfactory and trigeminal nerve pathways. Nonaka et al. (2008) showed that radiolabeled GALP reaches the brain parenchyma within 5–10 minutes after intranasal administration, with preferential accumulation in limbic regions including the vHPC.

Our intranasal administration results showed that AR−M1896 significantly ameliorated PS−induced anhedonia (restored sucrose preference). However, no significant improvement was observed in the forced swim test, a measure of despair−like passive coping behavior. This differential behavioral effect may be explained by regional differences in drug concentration achieved via intranasal versus direct intra−vHPC infusion. Whereas intra−vHPC infusion delivers a high local concentration (1 nmol/animal) directly to the target region, intranasal delivery likely produces lower and more diffuse vHPC concentrations, which may be sufficient to rescue anhedonia but insufficient to fully engage the vHPC receptor reserve required for despair−like behavior. Importantly, despite the less robust effect on passive coping, intranasal AR−M1896 significantly increased ATP content and upregulated PINK1 and Parkin expression in the vHPC, further confirming the protective effect of GalR2 activation on mitochondrial bioenergetics and mitophagy signaling in this region. The behavioral rescue by AR-M1896 was accompanied by reversal of ATP and PINK1/Parkin deficits, which strengthens the mechanistic interpretation. These data support an association that aligns with our proposed pathway. while definitive causality would require additional pathway-specific manipulations, such as blocking PINK1/Parkin signaling *in vivo*.

Collectively, these results indicate that intranasal delivery of AR−M1896 is a potentially informative pharmacological approach to ameliorate core depressive−like phenotypes induced by PS, with particularly robust effects on anhedonia. The complementary intra−vHPC infusion experiments further establish a causal role for vHPC GalR2 signaling in the observed mitochondrial rescue.

The *in vitro* experiments provide mechanistic insight into how GalR2 signaling may protect against glucocorticoid−induced mitochondrial damage. High−dose corticosterone in GalR2−expressing HEK293 cells recapitulated key pathological features seen in the vHPC of PS offspring. These features included decreased cell viability, reduced PINK1/Parkin expression, loss of mitochondrial membrane potential and mitochondrial fragmentation. These observations are consistent with previous reports that glucocorticoid excess impairs mitochondrial function, depolarizes mitochondrial membranes, and promotes oxidative stress and cell death (15). Pharmacological activation of GalR2 with AR−M1896 significantly attenuated these corticosterone−induced deficits and improved mitochondrial morphology. Conversely, these protective effects were abolished by co-application of the GalR2 antagonist M871. These data indicate that GalR2 activation enhances cellular resilience to glucocorticoid toxicity by preserving mitochondrial membrane potential and morphology, likely via maintaining or restoring PINK1/Parkin−dependent mitophagy.

At the signaling level, GalR2 is known to activate phospholipase C and downstream effectors such as PKC, ERK, and PI3K/Akt—pathways implicated in regulating mitochondrial dynamics, biogenesis, and autophagy. While this study did not directly dissect these specific cascades, the parallel rescue of PINK1/Parkin expression and mitochondrial integrity by GalR2 activation in both *in vivo* and *in vitro* models supports the existence of a functional GalR2–PINK1/Parkin–mitochondria axis. The intra−vHPC infusion data further strengthen this axis by demonstrating that local GalR2 activation is sufficient to elevate PINK1 and Parkin protein levels and ATP content (**Figure 4**), providing causal *in vivo* evidence for the GalR2–PINK1/Parkin pathway in the vHPC. Future studies are needed to determine the downstream mediators and whether GalR2 directly regulates PINK1/Parkin.

Several limitations should be considered. First, only male offspring were examined to avoid potential confounding by the estrous cycle, allowing a clearer dissection of the underlying molecular mechanisms. Future studies should include female offspring with careful monitoring of estrous cycle stage to evaluate possible sex differences in the GalR2–PINK1/Parkin–mitophagy axis and the antidepressant-like effects of AR−M1896. Second, we did not directly quantify AR−M1896 concentrations in brain tissue or assess its regional distribution following intranasal administration. Future studies should employ liquid chromatography–tandem mass spectrometry (LC−MS/MS) or radiolabeled tracer methods to measure brain drug levels and map regional exposure. Nevertheless, although intranasal delivery may not restrict drug distribution exclusively to the vHPC, our complementary intra−vHPC infusion experiments directly demonstrate that local GalR2 activation within the vHPC is sufficient to recapitulate both the molecular (ATP/PINK1/Parkin) and behavioral (amelioration of anhedonia and despair) effects observed with intranasal administration. This convergence of effects across delivery routes supports a central, vHPC−mediated mechanism of action. Third, *in vitro* experiments were performed in GalR2−expressing HEK293 cells rather than primary neurons; these findings provide receptor−specific mechanistic support but require validation in a native neuronal context. While the HEK293 system enabled clean receptor−specific dissection, we acknowledge that these kidney−derived cells do not fully recapitulate neuronal mitochondrial dynamics. Nonetheless, the consistency between our *in vitro* results and *in vivo* findings from the ventral hippocampus supports the translational relevance of the proposed GalR2–PINK1/Parkin–mitophagy axis. Future studies using primary neuronal cultures or neuron−specific GalR2 manipulations are warranted to confirm these mechanisms in a more physiologically relevant setting. Fourth, we did not directly test the necessity of PINK1/Parkin signaling for the behavioral rescue (e.g., via vHPC−specific knockdown); however, the strong correlation between local GalR2 activation, restoration of PINK1/Parkin and ATP levels, and behavioral improvement argues for functional relevance. Fifth, although AR−M1896 is a GalR2−selective agonist, its receptor occupancy and brain distribution after intranasal delivery remain to be fully characterized. Finally, we did not perform loss−of−function experiments (e.g., vHPC GalR2 knockdown) to test whether endogenous vHPC GalR2 is required for the effects of intranasal AR−M1896; this remains an important goal for future investigation.

From a psychiatric perspective, this work supports a model in which early environmental adversity produces enduring molecular and organelle-level alterations within stress- and reward-related circuits, thereby biasing individuals toward maladaptive affective states. By identifying a GalR2–mitochondrial axis in the ventral hippocampus, our study integrates neuropeptide signaling with mitochondrial homeostasis as a convergent pathway through which PS may shape emotional behavior across the lifespan. Notably, the complementary use of intra−vHPC infusion allowed us to establish regional causality, directly demonstrating that GalR2 agonism within the vHPC recapitulates the antidepressant−like and mitochondria−rescuing effects of systemic intranasal delivery. Although further studies are needed, targeting neuropeptide–mitochondrial interactions may represent a novel direction for mechanism-based interventions in stress-related mood disorders.

## Data Availability

The raw data supporting the conclusions of this article will be made available by the authors, without undue reservation.
